# The NAD-Dependent Deacetylase Sirtuin-1 Regulates the Expression of Osteogenic Transcriptional Activator Runt-Related Transcription Factor 2 (Runx2) and Production of Matrix Metalloproteinase (MMP)-13 in Chondrocytes in Osteoarthritis

**DOI:** 10.3390/ijms17071019

**Published:** 2016-06-28

**Authors:** Koh Terauchi, Hajime Kobayashi, Kanaka Yatabe, Naoko Yui, Hiroto Fujiya, Hisateru Niki, Haruki Musha, Kazuo Yudoh

**Affiliations:** 1 Department of Sports Medicine, St. Marianna University School of Medicine, Sugao 2-16-1, Miyamae-ku, Kawasaki 216-8511, Japan; koh.seikei@marianna-u.ac.jp (K.T.); h4kobayashi@marianna-u.ac.jp (H.K.); kanaka@marianna-u.ac.jp (K.Y.); aburainisuke@marianna-u.ac.jp (N.Y.); fujiya-1487@marianna-u.ac.jp (H.F.); musha@marianna-u.ac.jp (H.M.); 2 Department of Orthopaedic Surgery, St. Marianna University School of Medicine, Sugao 2-16-1, Miyamae-ku, Kawasaki 216-8512, Japan; h2niki@marianna-u.ac.jp; 3 Department of Frontier Medicine, Institute of Medical Science, St. Marianna University School of Medicine, Sugao 2-16-1, Miyamae-ku, Kawasaki 216-8512, Japan

**Keywords:** osteoarthritis, NAD-dependent deacetylase, sirtuin, Runx2, chondrocytes

## Abstract

Aging is one of the major pathologic factors associated with osteoarthritis (OA). Recently, numerous reports have demonstrated the impact of sirtuin-1 (Sirt1), which is the NAD-dependent deacetylase, on human aging. It has been demonstrated that Sirt1 induces osteogenic and chondrogenic differentiation of mesenchymal stem cells. However, the role of Sirt1 in the OA chondrocytes still remains unknown. We postulated that Sirt1 regulates a hypertrophic chondrocyte lineage and degeneration of articular cartilage through the activation of osteogenic transcriptional activator Runx2 and matrix metalloproteinase (MMP)-13 in OA chondrocytes. To verify whether sirtuin-1 (Sirt1) regulates chondrocyte activity in OA, we studied expressions of Sirt1, Runx2 and production of MMP-13, and their associations in human OA chondrocytes. The expression of Sirt1 was ubiquitously observed in osteoarthritic chondrocytes; in contrast, Runx2 expressed in the osteophyte region in patients with OA and OA model mice. OA relating catabolic factor IL-1βincreased the expression of Runx2 in OA chondrocytes. OA chondrocytes, which were pretreated with Sirt1 inhibitor, inhibited the IL-1β-induced expression of Runx2 compared to the control. Since the Runx2 is a promotor of MMP-13 expression, Sirt1 inactivation may inhibit the Runx2 expression and the resultant down-regulation of MMP-13 production in chondrocytes. Our findings suggest thatSirt1 may regulate the expression of Runx2, which is the osteogenic transcription factor, and the production of MMP-13 from chondrocytes in OA. Since Sirt1 activity is known to be affected by several stresses, including inflammation and oxidative stress, as well as aging, SIRT may be involved in the development of OA.

## 1. Introduction

In osteoarthritis (OA), mechanical and chemical stresses to articular cartilage downregulate chondrocyte activities and induce secretion of catabolic factors, such as proinflammatory cytokines and cartilage degrading enzymes, by chondrocytes [1,2,3,4]. In addition to mechanical and chemical stresses, chondrocyte aging is one of the major pathologic factors associated with OA. Indeed, Martin et al. reported that aging decreases the chondrocyte activity to maintain the homeostasis of articular cartilage tissue [5,6,7]. They examined different biomarkers of cellular aging in human articular cartilage tissues and found that chondrocyte senescence correlated with donor aging. Their findings clearly demonstrate that the association between OA and aging is due, at least in part, to the age-related decrease of chondrocytes and cartilage matrix function with aging. Aged chondrocytes may show an insufficient response to anabolic and catabolic factors, such as inflammation and oxidative stress, resulting in cartilage matrix degradation from unbalanced catabolic and anabolic activities [5,8,9,10,11,12,13,14].

Recently, numerous reports have demonstrated the impact of the NAD-dependent deacetylase sirtuin-1 (Sirt1) on human aging and age-related diseases [15]. It is well known that Sirt1, when adjusting the pattern of cellular metabolism to nutrient availability, can regulate many metabolic functions including DNA repair, genome stability, inflammatory response, apoptosis, cell cycle, and mitochondrial functions [15]. It has been demonstrated that Sirt1 insufficiency may induce the calcification and atherosclerosis in cardiovascular tissues, through a mechanism involving upregulation of Runt-related transcription factor 2 (Runx2), which is the osteogenic transcriptional activator [16]. This suggests that Sirt1 may protect against the vascular calcification and maintain the vascular function with aging. In contrast to this finding, several reports suggested that Sirt1 promotes the expression of Runx2, and the resultant osteogenic transcription and chondrogenic differentiation of mesenchymal stem cells [17,18]. There are still some conflicting opinions concerning the association between Sirt1 and Runx2 expression. In addition, although there is a general consensus regarding the involvement of Runx2, which is required for chondrocyte hypertrophy and osteophyte formation, in the pathogenesis of OA [19], the role of Sirt1 in the Runx2 in OA chondrocytes remains unknown.

We postulated that the NAD-dependent deacetylase Sirt1 regulates the development of chondrocyte hypertrophic lineage and the progression of cartilage degeneration through activation of Runx2 and production of matrix metalloproteinase (MMP)-13 by chondrocytes in OA. Runx2 is well known to be a promotor of the activation of MMP-13 in chondrocytes [20,21]. If Sirt1 influences Runx2 expression, MMP-13 production could also be mediated by Sirt1 in OA chondrocytes.

To verify whether or not Sirt1 regulates chondrocyte activity in OA, we examined expressions of Sirt1 and Runx2 and production of MMP-13, and their correlations in human chondrocytes.

## 2. Results and Discussion

### 2.1. Expression of Runx2 in Human Chondrocytes from Patients with OA

**Figure 1:**
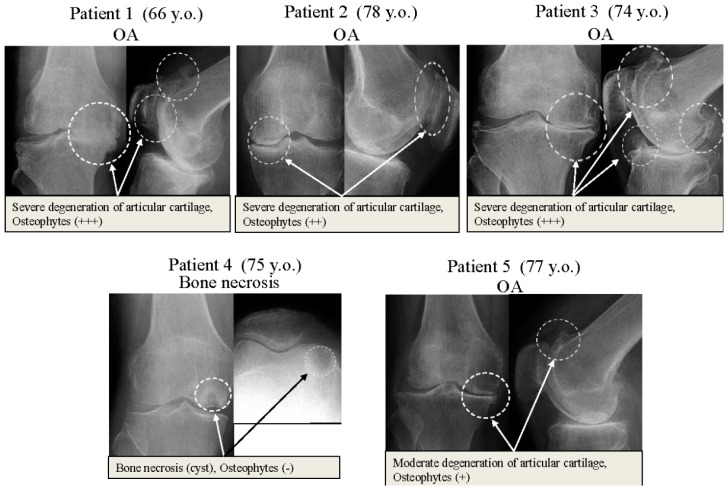
X-ray images of knee joints of donor patients. Patient 1; female, 66 years of age, Patient 2; female, 78 years of age, Patient 3; male, 74 years of age, Patient 4; female, 75 years of age, Patient 5; female, 77 years of age. Patient 1, 2, 3 had severe knee OA with severe cartilage degeneration and osteophyte formation. Patient 4 had idiopathic osteonecrosis of femoral condyle. Patient 5 had moderate knee OA with cartilage degeneration and osteophyte formation. OA; osteoarthritis, y.o.; years old.

**Figure 2:**
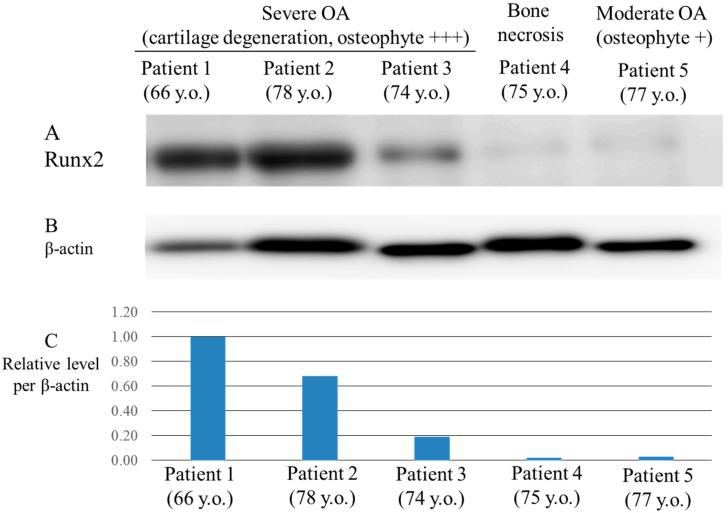
Expression of Runx2 in osteoarthritic chondrocytes from donors. (**A**) Expression of Runx2 in chondrocytes from patients with OA (Patient 1, 2, 3, 5) and from patient with idiopathic osteonecrosis of femoral condyle (Patient 4); (**B**) Expression of β-actin.; (**C**) Relative ratio against the level of expression of β-actin. The level of Runx2 expression was analyzed as a relative ratio against the level of expression of β-actin. Runx2 protein was strongly expressed in osteoarthritic chondrocytes from “Patient 1”, “Patient 2” and “Patient 3” in comparison with “Patient 4” and “Patient 5”.

The clinical features of patients we studied are summarized in Table 1. The pre-operative X-ray features of patients are shown in Figure 1. Runx2 was expressed by osteoarthritic chondrocytes from patients with severe OA. As shown in Figure 2A, the Runx2 protein was strongly expressed in osteoarthritic chondrocytes from “Patient 1”, “Patient 2” and “Patient 3”. These three patients with OA had severe cartilage degeneration and osteophyte formations (Table 1, Figure 1).

The expression of Runx2 in chondrocytes from “Patient 5” with a moderate degree of cartilage degeneration and osteophyte formation was lower than those from other patients with severe OA and osteophyte formation (Figure 1). In contrast to OA patients, the level of expression of Runx2 was very weak in chondrocytes from patient “Patient 4” who had bone necrosis of the femoral condyle (Figure 2A). The degree of Runx2 expression in chondrocytes was associated with the severity of cartilage degeneration and osteophyte formation in articular cartilage tissues. Previous reports demonstrated that induction of an osteogenic transcription factor Runx2 in chondrocytes contributed to the progression of OA through the acceleration of chondrocyte hypertrophy [19,21]. The association of Runx2 expression with the severity of OA including osteophyte formation found in our study is consistent with the results of these reports.

### 2.2. OA-Related Factor IL-1β Induces Runx2 Expression in Human Osteoarthritic Chondrocytes

**Figure 3:**
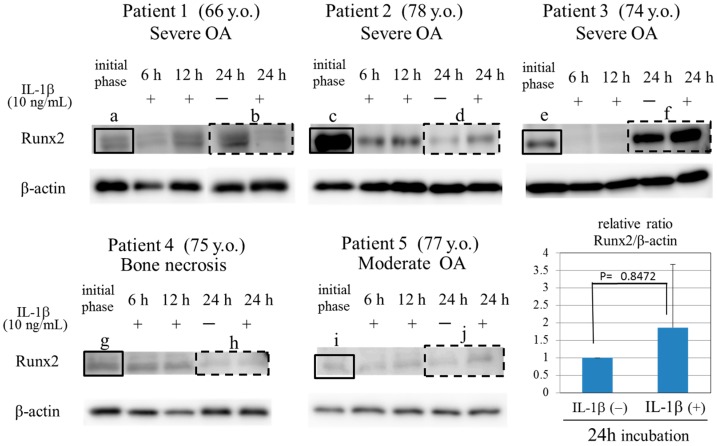
Effects of IL-β on Runx2 expression in osteoarthritic chondrocytes from donor patients. Time course of Runx2 expression in chondrocytes in the presence of IL-1β (10 ng/mL). At the initial phase, the level of Runx2 expression in chondrocytes was higher in patients with severe OA (Patient 1 (**a**), Patient 2 (**c**), and Patient 3 (**e**)) than in patient with bone necrosis (Patient 4 (**g**)) and patient with moderate OA (Patient 5 (**i**)). After 24 h-incubation period, the expression level of Runx2 increased in the presence of the IL-1β (10 ng/mL) in chondrocytes from Patient 2 (**d**), Patient 3 (**f**), Patient 5 (**j**) but not Patient 1 (**b**) and Patient 4 (**h**), although its expression levels decreased transiently at 6 and 12 h incubation periods. Solid box indicates the expression of Runx2 at the initial phase. The dash line box indicates the expression of Runx2 at 6 and 12 h incubation periods. The treatment with IL-1β trended to induce the expression of Runx2, although no significant difference was observed between the IL-1β-treated group and the control group.

Osteoarthritic chondrocytes from patients with severe OA (Patients 1–3) expressed Runx2 protein during the initial phase (Figure 3a,c,e). These three patients with OA all exhibited severe cartilage degeneration and osteophyte formation (Table 1, Figure 1). The expression of Runx2 in chondrocytes from a patient with moderate OA (Patient 5) and from a patient with bone necrosis of the femoral condyle (Patient 4) were lower than those from other patients with severe OA (cartilage degeneration and osteophyte formation) at the initial phase (Figure 3g,i). This expression pattern of Runx2 in chondrocytes coincided with the finding shown in Figure 2A. These findings indicate that the Runx2 is implicated with the OA progression, and especially osteophyte formation, in OA cartilage.

After a 24 h-incubation period, the expression level of Runx2 increased in the presence of the OA-related factor (IL-1β, 10.0 ng/mL), in chondrocytes from Patients 2, 3, and 5 (Figure 3d,f,j) but not Patient 1 (Figure 3b), although its expression levels decreased transiently at the 6 and 12 h time-points. The evidence for an IL-1β-induced increase in Runx2 in OA chondrocytes also indicates the role of Runx2 in the development of OA. However, in the present study, there was donor variation of clinical features from the point of view of osteoarthritic degeneration of articular cartilage, especially osteophyte formation, even though all patients underwent arthroplastic surgery. The variation of clinical features was summarized in Table 1.

In addition, the time course of Runx2 expression in the IL-1β-treated chondrocytes was varied in OA patients. There is a general consensus that proinflammatory cytokine, IL-1β, is an OA related factor. Treatment with IL-1β may affect the chondrocyte microenvironment, viability and several cellular activities including the Runx2 transcription. Further studies are needed to verify why the time courses of Runx2 expression were different in OA patients. IL-1β-induced change of chondrocyte microenvironment or different responsibility to IL-1β in each patient might be implicated in donor variation. The donor variation may affect the expression patterns of Runx2 in Figure 2 and Figure 3.

### 2.3. Immunopositivity of Runx2 and Sirt1 in the Articular Cartilage in OA Model Mouse

To clarify whether the expression of Runx2 and Sirt1 was correlated with degeneration of articular cartilage in OA, the immunopositivity for Runx2 and Sirt1 was examined in the degenerated articular cartilage sections from a spontaneous OA model mouse, which develops an osteoarthritic process (STR/OrtCrlj mouse). For immunohistochemical analysis, we got whole knee joint tissues (juxta-articular bone, subchondral bone, meniscus, ligaments, and cartilage tissues) from the OA mouse model. Using the OA mouse model, we can observe the progression of degeneration of OA joint tissues, not only cartilage but also juxta-articular bone and subchondral bone. In elder mice, osteophytes were expressed at the juxta-articular lesion in OA joints, and grew gradually with advance of mouse aging. Interestingly, the expression of Runx2 was localized at the osteophytic lesion in OA mice. In contrast, not only osteophytes but also Runx2 were undetectable in younger mice.

**Figure 4:**
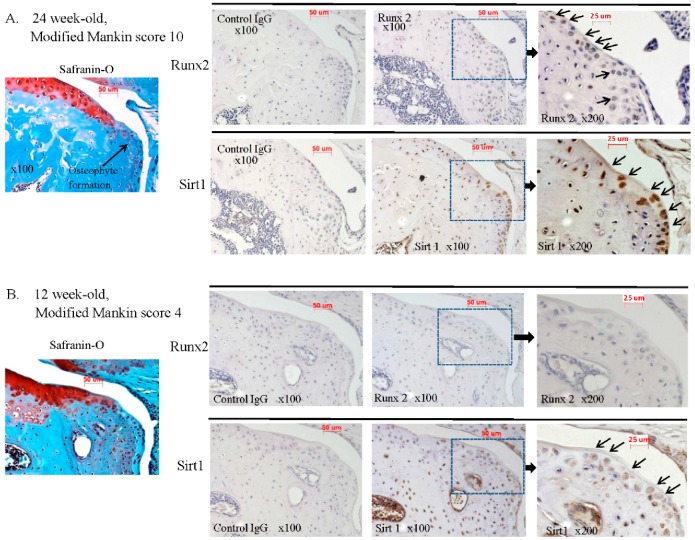
Representative images for Runx2- and Sirt1-immunostaining in mouse OA articular cartilage. (**A**) Representative images of Safranin O staining and Runx2- and Sirt1-immunohistologic staining in severe OA articular cartilage from 24-week old STR/OrtCrlj mice; Safranin O staining (×100), Runx2 immunostaining (×100, ×200), Sirt1 immunostaining (×100, ×200). A higher expression pattern of Runx2 was observed at the osteophyte region in the severely degenerated cartilages. Arrows indicate the Runx2- or Sirt1-immunopositive chondrocytes; (**B**) Representative images of Safranin O staining and Runx2- and Sirt1-immunohistologic staining in moderate OA articular cartilage from 12-week old STR/OrtCrlj mice; Safranin O staining (×100), Runx2 immunostaining (×100, ×200), Sirt1 immunostaining (×100, ×200). Almost of the mildly degenerated cartilages were negative for Runx2 immunostaining in 12-year old mice. Sirt1 was ubiquitously detected in chondrocytes of both normal articular cartilages and degenerated articular cartilages, regardless with or without osteophyte formation. Arrows indicate the Runx2- or Sirt1- immunopositive chondrocytes.

Figure 4 shows representative images of articular cartilages (Safranin-O staining) and immunohistologic staining of Runx2 and Sirt1 in articular cartilages of OA mice. The level of cartilage degeneration (modified Mankin score) and the immunopositivity of Runx2 and Sirt1 in samples of OA mouse articular cartilage are summarized in Table 2.

Severe degeneration of articular cartilage, such as decreased level of Safranin-O staining, chondrocyte loss, surface irregularities, superficial clefts and osteophyte formation were observed in degenerated articular cartilage from 24-week old mice (representative image in Figure 4A; modified Mankin score 10, Table 2). Interestingly, many strongly Runx2-immunopositive chondrocytes were detected in the osteophyte regions of degenerated articular cartilage from older 24-week-old mice (representative image in Figure 4A, Table 2). In contrast, only faint staining of Runx2 was observed in both normal articular cartilage and slightly degenerated articular cartilage with no osteophyte formation in 12-old mice (Figure 4B, Table 2). In 20-week-old mice, osteophytes were observed at the attachment region of the anterior cruciate ligament (ACL) (data not shown). Around the ACL, we also observed Runx2 protein expression in chondrocytes. Twelve-week old mice showed moderate cartilage degeneration with no osteophyte formation (Figure 4; modified Mankin score 4B, Table 2).

We found that articular cartilage, which had no osteophytes, showed no immunopositivity for Runx2 in chondrocytes (5 osteophyte regions per mouse, 3 mice). These findings indicate that expression of Runx2 in chondrocytes is correlated with osteophyte formation in OA. Our histologic findings indicate that the expression of Runx2 is correlated with osteophyte formation.

In contrast to the expression pattern of Runx2 in articular cartilage, Sirt1 was ubiquitously detected in chondrocytes of both normal and degenerated articular cartilage, regardless of whether or not osteophyte formation was present (Figure 4A,B, Table 2). At the osteophyte region in degenerated articular cartilage, most Sirt1-positive chondrocytes expressed the Runx2 protein (mean percentage of immunopositivity for Runx2 in Sirt1 positive cells: 92%), although chondrocytes in degenerated cartilage with no osteophytes expressed only Sirt1, but not Runx2 (Figure 4A). These findings suggest that Runx2 expression is associated with Sirt1, at least in part, at the osteophyte region. Recently, several reports demonstrated that Sirt1 plays an important part in chondrocyte activity and cartilage metabolism [22,23,24]. Dvir-Ginzberg et al. demonstrated that Sirt1 regulated the expression of cartilage-specific gene in human chondrocytes [22]. Takayama et al. reported the role of Sirt1 in the regulation of human chondrocyte apoptosis [23]. In addition, it has been reported that Sirt1 may promote the chondrogenic differentiation of mesenchymal stem cells [18], suggesting the potential involvement of Sirt1 in the chondrocyte differentiation and pathogenesis of OA.

Thus, we postulated that Sirt1 might have the potential to regulate Runx2 expression in chondrocytes in OA. Since Runx2 regulates the expression of MMP-13 in chondrocytes [19,20], Sirt1 may also regulate the MMP-13 production by chondrocytes through the modulation of Runx2 in OA.

### 2.4. Sirt1 Regulates Runx2 Expression and MMP-13 Production in OA Chondrocytes

**Figure 5:**
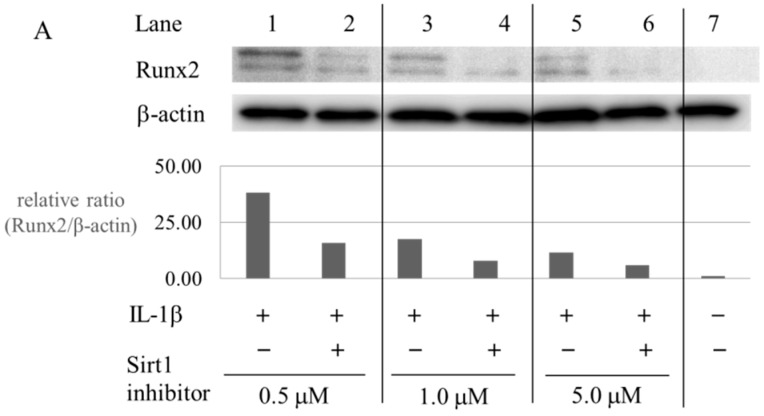

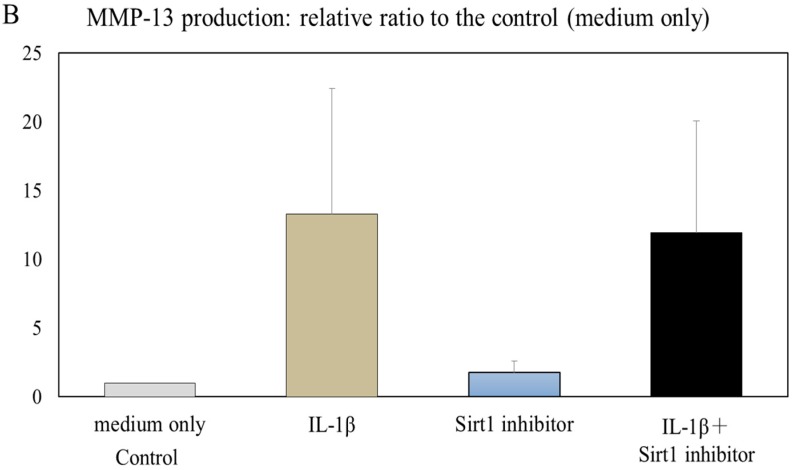
Sirt1 regulates Runx2 expression and MMP-13 production in osteoarthritic chondrocytes. To address whether Sirt1 is necessary to express the Runx2 protein in chondrocyte, OA chondrocytes were preincubated with Sirt1 inhibitor (S)-35 (0.5, 1.0, 5.0 μM). (**A**) In OA chondrocytes, treatment with IL-1β stimulated the expression of Runx2 (lane 1, 3, 5) compared to the control (lane 7). Sirt1 inhibitor inhibited the IL-1β-induced acceleration of Runx2 expression in chondrocytes (lane 2, 4, 6); (**B**) Treatment with IL-1β significantly accelerated MMP-13 production from chondrocytes in comparison with the control (medium only) (*p* = 0.037). Treatment with Sirt1 inhibitor only did not influence the MMP-13 production from chondrocytes in comparison with the control. The inhibition of Sirt1 with Sirt1 inhibitor trended to inhibit the IL-1β-accelerated production of MMP-13 in chondrocytes, although no significant difference was observed between the Sirt1 inhibitor-treated IL-1β-treated chondrocytes and the Sirt1 inhibitor + IL-1β-treated chondrocytes (*p* = 0.832). The levels of MMP-13 production in both Sirt1 inhibitor-treated group and control group were lower than in those IL-1β-treated groups.

To address whether Sirt1 modulates the expression of Runx2 and MMP-13 in chondrocytes, chondrocytes from four OA patients were pre-treated with Sirt1 inhibitor and then cellular activities were examined in vitro (Figure 5).

In the absence of IL-1β, the level of Runx2 expression in chondrocytes was very weak (Figure 5A, lane 7). In contrast, treatment with IL-1β stimulated the Runx2 expression in chondrocytes (Figure 5A, lane 1, 3, 5). Interestingly, pre-treatment with Sirt1 inhibitor (S)-35 (0.5, 1.0, 5.0 μM) inhibited the IL-1β-induced increased in Runx2 expression in chondrocytes (Figure 5A, lane 2, 4, 6). Our data clearly indicated that osteoarthritic chondrocytes, which were pre-treated with Sirt1 inhibitor, expressed the decreased level of Runx2 even in the presence of IL-1β (Figure 5A). Buhrmann et al. demonstrated that the Sirt1 activator, resveratrol, induced expression of cartilage-specific genes including Runx2 in mesenchymal stem cells [18]. From their results, they concluded that Sirt1 promotes chondrogenic differentiation of mesenchymal stem cells through the modulation of Runx2 [18]. Kim et al. also indicated that treatment with resveratrol significantly upregulates Sirt1 gene expression in normal and osteoarthritic chondrocytes and induces chondrocytes to differentiate into a hypertrophic state through upregulation of collagen type I, collagen type X and Runx2 [25]. It has been reported that Runx2 regulates the expression of MMP-13 in chondrocytes [19,20] These data led to our hypothesis that Sirt1 might activate Runx2 in osteoarthritic chondrocytes, resulting in the upregulation of MMP-13.

As shown in Figure 5B, treatment with IL-1β significantly increased the MMP-13 production from chondrocytes in comparison with controls (medium only) (*p* = 0.037). Pre-treatment with the Sirt1 inhibitor (S)-35 tended to reduce the IL-1β-accelerated production of MMP-13 in chondrocytes, although no significant difference was observed between the IL-1β-treated chondrocytes and the Sirt1 inhibitor + IL-1β-treated chondrocytes (*p* = 0.832, Figure 5B). NAD-dependent deacetylase Sirt1 can act on cell activation and many cellular metabolisms as an important regulator; cell cycle, DNA repair, genome instability, mitochondrial functions, cell differentiation, inflammatory response, and anti-apoptotic pathways, etc. in a variety of cells [15]. Generally, it has been demonstrated that Sirt1 is ubiquitously expressed human somatic cells and controls many cellular activities. In addition, it is well known that many stresses to the cells, such as oxidative stress, inflammation, or aging, affect the Sirtuin1 activity [15,17,18]. The stress-induced inactivation of Sirt1 may reveal the cell in activation and downregulation of cellular metabolism. Since mechanical and chemical stresses to articular cartilage and chondrocytes are known to induce the development of OA, these extrinsic stresses to chondrocytes may influence the Sirt1 activity in chondrocytes and resultant dysfunction of chondrocytes. Thus, we postulated that the OA-related factor-induced change of Sirt1 activity may be involved in some changes of chondrocyte activity and metabolism during the progression of OA. In the current study, Sirt1 inactivation with Sirtuin1 inhibitor reduced the IL-1β-accelerated expression of Runx2 in OA chondrocytes, suggesting that the NAD-dependent deacetylase Sirtuin1 may regulate the osteogenic transcriptional activator Runx2 in chondrocytes. Osteogenic transcription factor Runx2 in chondrocytes is known to be closely involved in the endochondral ossification in articular cartilage tissue. Previously, several reports demonstrated that Sirt1 promotes the expression of Runx2 and the resultant osteogenic and chondrogenic differentiation in a variety of cells [16,17,18]. Iijima suggested that Sirt1 promotes the Runx2 activity via a P53/P21 pathway in vascular endothelial cells [16]. Iijima concluded that Sirtuin1 promotes the vascular calcification via the Runx2 pathway. There may be some common mediators (such as P53 or P21) in the Sirt1-associated pathway for expressions of Runx2 in vascular endothelial cells. In addition, in human chondrocytes, Sirt1 is one of important mediators to regulate the expression of Runx2 through the mechanism involving the acceleration or reduction of signal transduction pathways. There may be some common mediators in the Sirt1-associated pathways for expressions of MMP-13 and Runx2 in OA chondrocytes.

Our data suggest that Sirt1 inactivation may inhibit the expression of Runx2 in OA chondrocytes. Since there is a general consensus that the Runx2 is a promotor of the activation of MMP-13 in chondrocytes [20,21], therefore, we would like to conclude that the Sirtuin1 may, at least in part, have a potential to regulate the expression of MMP-13 through the mechanism involving the Sirtuin1-regulated Runx2 expression in chondrocytes, although further studies are required to clarify the exact effect of Sirtuin1 on Runx2 and MMP-13 expressions.

**Figure 6:**
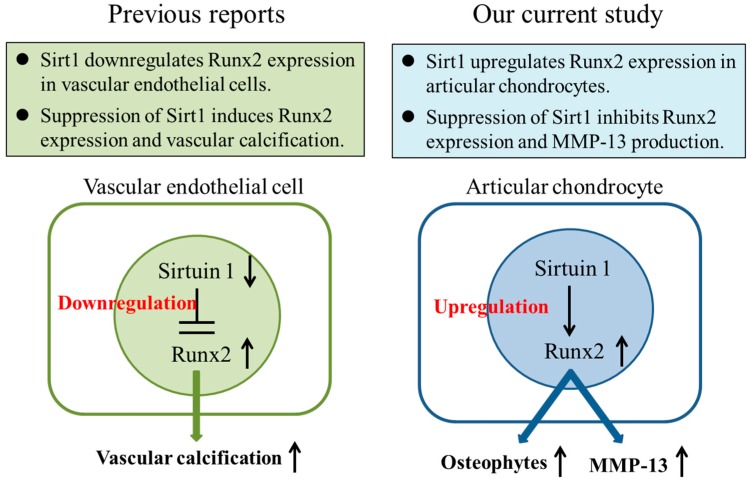
Summary of our current study. The NAD-dependent deacetylase Sirt1 may regulate the osteogenic transcription and chondrocyte hypertrophic factor Runx2 and matrix degrading enzyme MMP-13 in chondrocytes in OA. Arrow “↑” indicates upregulation, Arrow “↓” indicates downregulation.

In contrast to our findings, several other reports demonstrated that Sirt1 downregulated chondrocyte gene expression in OA [23,24]. Fujita et al reported that overexpression of Sirt1 by gene transfection in chondrocytes inhibited expression of osteoarthritic genes such as MMP-13 [24]. They also demonstrated that reduction of Sirt1 by small-interfering RNA caused an increase in MMP-13 production from chondrocytes, suggesting the down-regulatory effect of Sirt1 on the production of MMP-13in chondrocytes. These findings suggest that Sirt1 plays a protective part to suppress IL-1β-induced expression of cartilage degrading enzyme, MMP-13, partially through the NF-kB pathway. However, they did not mention whether or not Sirt1 modulates the expression of Runx2, which is a key regulator of MMP-13 expression, in chondrocytes. In the current study, our findings provided evidence that Sirt1 inactivation inhibits the expression of Runx2 and IL-1β-stimulated MMP-13 in OA chondrocytes. Buhrmann et al. and Kim et al. also indicated that Sirt1 activation induced expression of cartilage-specific genes including Runx2 in mesenchymal stem cells and chondrocytes, respectively, suggesting the correlation between the Sirt1 activity and the Runx2 activity [18,25]. Although further studies are needed to verify the association between Runx2 and Sirt1 in chondrocytes, Sirt1 may play important roles in chondrocyte hypertrophy/osteophyte formation and MMP-13 expression through activation of Runx2 in chondrocytes (Figure 6).

## 3. Experimental Section

### 3.1. Chondrocyte Isolation and Cell Culture

Human articular cartilage tissues of the knee joints were obtained from five patients who underwent arthroplastic knee surgery, after obtaining informed consent. Four of the patients were treated for OA (3 females aged 66, 75, and 77 years, and 1 male aged 74 years) and one for idiopathic osteonecrosis of the femoral condyle (female aged 75 years). The protocol of this study was accepted by our University ethical committee in St. Marianna University School of Medicine (permission code: No.1315; date: 7 January 2006). The clinical features of patients are summarized in Table 1. The pre-operative X-ray features of patients are shown in Figure 1.

At the surgery, degenerated bone and articular cartilage tissues were widely resected for prosthetic joint replacement. Articular cartilage tissues were carefully collected from the resected bone and cartilages (circled areas in each patient, Figure 1). Cartilage samples were cut into small pieces and then, washed with phosphate-buffered saline (PBS). Cartilage pieces were digested with 1.5 mg/mL collagenase B (Sigma, St. Louis, MO, USA) in Dulbecco’s modified Eagle’s medium (DMEM) (Sigma) at 37 °C overnight on a shaking platform. The isolated chondrocytes were centrifuged and washed three times with PBS. In addition, chondrocytes were cultured in DMEM supplemented with 10% heat-inactivated fetal calf serum, 2 mM l-glutamine, 25 mM HEPES (2-(4-(2-hydroxyethyl)-1-piperazinyl) ethanesulfonic acid), and 100 U/mL penicillin and streptomycin at 37 °C in a humidified atmosphere of 95% air and 5% CO_2_ [26].

### 3.2. Immunohistochemistry for Runx2 and Sirt1 in Mouse OA Articular Cartilages

To clarify the involvement of Runx2 and Sirt1 in the development of cartilage degeneration, we used immunohistochemical analysis to investigate levels of expression of Runx2 and Sirt1 in chondrocytes from an OA mouse model (STR/OrtCrlj mouse). Twenty-four STR/OrtCrlj mice (male, 12 weeks of age) were purchased from Charles River Japan (Yokohama, Japan) [27]. The mice were euthanized with diethyl ether at 4, 8, 12, or 16 weeks from the start of the study (*n* = 6/time-point). Articular cartilage samples with the subchondral bone were fixed with 4% paraformaldehyde solution for 2 days, and then decalcified in 4% paraformaldehyde containing 0.85% sodium chloride and 10% acetic acid. The tissues were dehydrated in a series of ethanol solutions, infiltrated with xylene and embedded in paraffin. The samples were cut into 6-μm sections. For histological examination of the degree of degeneration in the articular cartilage tissues, some sections were stained with Safranin O-fast green to determine the level of proteoglycan expression. Each cartilage sample was evaluated histologically for the degree of degeneration according to the modified Mankin score (Table 3) [26,28]. To determine the mean damage score, two to three regions were examined from the each animal. Three independent observers examined cartilage damage in a blinded manner.

For immunostaining of articular cartilage, serial sections of paraffin-embedded bone and cartilage tissues were immunostained with antibodies for Runx2 or Sirt1. The 6-μm sections were treated with 3% H_2_O_2_, followed by blocking of nonspecific protein binding using a blocking agent (Protein Block, Dako, Carpinteria, CA, USA). The sections were incubated with polyclonal antibodies against Runx2 (#12556, 1:50 dilution; Cell Signaling Technology, Danvers, MA, USA) or Sirt1 (#SAB2501994, 1:200 dilution; Sigma) for 1 h at room temperature. The sections were then followed by incubation with biotinylated anti-mouse IgG (Dako, rabbit IgG X0936 for anti-Runx2 antibody (1:3000 dilution), or goat serum X0907 for anti-Sirt1 antibody (1:100 dilution)) for 30 min at room temperature. The sections were then incubated with a streptavidin-horseradish peroxidase complex (LSAB2 Kit; Dako) for 30 min at room temperature. Diaminobenzidine (Sigma) was used as a visible peroxidase reaction product. Finally, the sections were counterstained with Mayer’s haematoxylin (Sigma).

The number of Runx2- or Sirt1-positive cells was counted in degenerated articular cartilage and osteophyte regions under high-power magnification (×100) for each case [26].

### 3.3. Effects of IL-1β on Expression of Runx2 and Sirt1 Expressions in Chondrocytes.

To study the effects of the OA-related catabolic factor, IL-1β, on the Runx2 and Sirt1 expressions in chondrocytes, chondrocytes were incubated in the presence or absence of IL-1β (10.0 ng/mL) for 6, 12, or 24 h at 37 °C in a humidified atmosphere of 95% air and 5% CO_2_. After harvesting, protein was isolated from the cultured cells for immunoblotting analyses as previously described.

Expression levels of Runx2 and Sirt1 in OA chondrocytes were analyzed by western blotting. The antibodies used in the current study were polyclonal antibody against human Runx2 (#FA2006, 1:500 dilution; R&D Systems Inc., Minneapolis, MN, USA), polyclonal antibody against human Sirt1 (1:2000 dilution; Abcam Inc., Cambridge, UK), β-tubulin (1:1000 dilution; Santa Cruz Biotechnology, Santa Cruz, CA, USA) and the corresponding secondary antibodies conjugated with horseradish peroxidase (Dako, goat IgG #p0449 for anti-Runx2 antibody (1:10,000 dilution), or rabbit IgG p04 for anti-Sirt1 antibody (1:10,000 dilution)). The western blotting was triplicated using same donor samples. The antibody-bound protein bands were visualized by an extended cavity laser system (GE Healthcare Bio-sciences KK, Tokyo, Japan). Densitometry of the signal bands was analyzed as described previously [26].

### 3.4. Effects of Sirt1 Inactivation on Runx2 Expression and MMP-13 Production in OA Chondrocytes

To verify the effects of Sirt1 inactivation on chondrocyte activity, we examined levels of the cartilage degrading enzyme MMP-13 and the osteogenic transcription factor Runx2 in Sirt1 inhibitor-treated chondrocytes in vitro. OA chondrocytes were obtained from OA patients who had undergone arthroplastic knee surgery (*n* = 4; all females, aged 72, 79, 75, 75 years).

Cultured chondrocytes were divided into 4 groups; control (medium only), IL-1β-treated group, Sirt1 inhibitor-treated group, and IL-1β + Sirt1 inhibitor-treated group. In the Sirt1 inhibitor-treated group and IL-1β + Sirt1 inhibitor-treated group, chondrocytes were pre-treated with Sirt1 inhibitor (S)-35 (#sc-204279, 0.5, 1.0, 5.0 μM, Santa Cruz Biotechnology Inc.) for 6 h at 37 °C in a humidified atmosphere of 95% air and 5% CO_2_. Then IL-1β (10.0 ng/mL) was added into the cell culture. The cells were returned to the incubator for a further 18 h. After the incubation period, the conditioned culture medium was collected and stored at −80 °C until analysis.

The levels of MMP-13 produced by chondrocytes were analyzed using an ELISA kit (MMP-13 assay kit; Amersham Biosciences, Little Chalfont, UK). The level of Runx2 expression in chondrocytes was examined by western blotting analysis as described above.

### 3.5. Statistical Analysis

The results for each experiment were determined from the mean of triplicated experiments. Date were expressed as mean ±standard deviation. For parametric date, the *t-*test was used to study the significance of differences between two groups. Non-parametric date were analyzed by Mann-Whitney *U* test. Probability values of <0.05 were considered statistically significant.

## 4. Conclusions

In conclusion, our data suggest that the NAD-dependent deacetylase Sirt1 may regulate the osteogenic transcription and chondrocyte hypertrophic factor Runx2 and cartilage matrix degrading enzyme MMP-13 in chondrocytes in OA. Since it has been already demonstrated that Sirt1 activity is influenced by several stresses, including inflammation and oxidative stress, as well as aging, SIRT may participate in the progression of OA. Our study may reveal a novel pathogenic mechanism linking the NAD-dependent deacetylase Sirt1 and its modulation of Runx2 in OA.

## Figures and Tables

**Table 1:** The clinical characteristics of patients.

Patients Number	Age/Gender	Grade of Cartilage Degeneration	Duration of Disease	Synovitis	Osteophyte Formation	Dysfunction of Joint	Treatment
① OA	66 y.o.	severe OA	~10 years	−	+++	severe	NSAIDs
	female						HA injection
② OA	78 y.o.	severe OA	>20 years	+	+++	severe	NSAIDs
	male						HA injection
③ OA	74 y.o.	severe OA	>20 years	−	+++	severe	NSAIDs
	female						HA injection
④ Bone necrosis	75 y.o.	moderate OA	~10 years	−	+	moderate	NSAIDs
	female						
⑤ OA	77 y.o.	moderate OA	>20 years	−	+	moderate	NSAIDs
	female						HA injection

y.o.: years old; NSAIDs: nonsteroidal anti-inflammatory drugs; HA: hyaluronic acid.

**Table 2:** Summary of immunohistologic analysis in OA mouse articular cartilages.

	24-Week Old Osteophyte+	24-Week Old Osteophyte+	24-Week Old Osteophyte−	20-Week Old Osteophyte+	16-Week Old Osteophyte−	12-Week Old Osteophyte−
Modified Mankin score	10	9	10	6	4	4
Runx2 positive (% per total cells)	15.1 ± 2.7	18.2 ± 1.8	N.A.	23.4 ± 3.1	5.4 ± 1.0	0.3 ± 0.6
Sirt1 Positive (% per total cells)	43.2 ± 14.2	N.A.	39.3 ± 6.5	65.8 ± 10.9	16.7 ± 7.3	33.3 ± 11.2

N.A.: not available.

**Table 3:** Modified Mankin Score (criteria for histological evaluation).

**Safranin O-fast green staining**	
0	uniform staining throughout articular cartilage
1	loss of staining in the superficial zone for less than one-half of the length of the plateau
2	loss of staining in the superficial zone one-half
3	loss of staining in the superficial and middle zones for less than one-half of the length of the plateau
4	loss of staining in the superficial and middle zones for one-half or more of the length of the plateau
5	loss of staining in all 3 zones for less than one-half of the length of the plateau
6	loss of staining in all 3 zones for one-half or more of the plateau
**Structure**	
0	normal
2	surface irregularities
3	>3 superficial clefts
4	1–3 clefts extending into the middle zones
5	>3 clefts extending into the middle zones
6	1–3 clefts extending into the deep zones
7	>3 clefts extending into the deep zones
8	clefts extending to calcified cartilage
**Chondrocyte loss**	
0	no decrease in cell
1	minimal decrease in cells
2	moderate decrease in cells
3	marked decrease in cells
4	very extensive decrease in cells

## Abbreviation

OA	Osteoarthritis
ROS	Reactive oxygen species
PBS	Phosphate-buffered saline
DMEM	Dulbecco’s modified Eagle’s medium
HEPES	2-[4-(2-hydroxyethyl)-1-piperazinyl] ethanesulfonic acid
IL-1β	Interleukin-1β
Runx2	Runt-related transcription factor 2
MMP-13	Matrix metalloproteinase-13
